# Blood circulation effect of fermented citrus bioconversion product (FCBP) in EA.hy926 endothelial cells and high-fat diet-fed mouse model

**DOI:** 10.29219/fnr.v68.10682

**Published:** 2024-11-07

**Authors:** Eun-Chae Cho, Hyuck Se Kwon, Na Young Lee, Hyun Jeong Oh, Yean-Jung Choi

**Affiliations:** 1 Department of Convergence Science, Sahmyook University, Seoul, Republic of Korea; 2 R&D Team, Food & Supplement Health Claims, Vitech Co., Ltd., Wanju, Republic of Korea; 3 Department of Food and Nutrition, Sahmyook University, Seoul, Republic of Korea

**Keywords:** fermented citrus bioconversion product, cardiovascular health, high-fat diet, endothelial function, bioactive flavonoids

## Abstract

**Background:**

The escalating global burden of cardiovascular diseases, largely driven by unhealthy lifestyle choices and dietary patterns, has intensified the search for effective and safe interventions. With current treatments often marred by significant side effects, the exploration of natural compounds such as flavonoids presents a compelling alternative.

**Objective:**

This study investigated the effects of fermented citrus bioconversion product (FCBP), a fermented citrus bioflavonoid, on various markers of cardiovascular health in the context of a high-fat diet.

**Design:**

*In vivo*, a high-fat diet-induced mouse model was used to assess the effects of FCBP on body weight, serum nitric oxide (NO) levels, activated partial thromboplastin time (aPTT), phosphatidylserine (PS) exposure on red blood cells, and the expression of inflammatory markers Intercellular Adhesion Molecule (ICAM)-1 and Vascular Cell Adhesion Molecule (VCAM)-1 in the thoracic aorta. *In vitro*, EA.hy926 endothelial cells were used to evaluate the compound’s effects on cell viability, NO production, endothelial nitric oxide synthase (eNOS) expression, and cell adhesion molecule (CAM) levels to further understand the mechanisms behind the *in vivo* findings.

**Results:**

*In vivo*, FCBP supplementation led to a dose-dependent reduction in weight gain, a significant decrease in serum NO levels at 10 mg/kg, and reduced ICAM-1 and VCAM-1 expressions in the thoracic aorta, indicating anti-inflammatory properties. PS exposure on red blood cells was also reduced, suggesting decreased procoagulant activity, while aPTT remained unchanged. *In vitro*, FCBP was non-cytotoxic to endothelial cells, showed a trend toward increased NO production and eNOS expression, and reduced the expression of ICAM-1 and VCAM-1, supporting its potential anti-inflammatory effects.

**Conclusions:**

FCBP demonstrates potential as a bioactive compound for managing cardiovascular health by reducing inflammation, mitigating weight gain, and influencing blood circulation-related parameters under high-fat diet conditions. Further studies, including diverse models and human trials, are warranted to elucidate its mechanisms and compare its efficacy with established cardiovascular therapeutics.

## Popular scientific summary

- This study investigated the effects of fermented citrus bioconversion product (FCBP), a fermented citrus bioflavonoid, on cardiovascular health in mice fed a high-fat diet and in endothelial cells.
- FCBP reduced weight gain and inflammatory markers (ICAM-1, VCAM-1) in mice.
- It decreased procoagulant activity without affecting normal blood clotting time.
- In endothelial cells, FCBP increased nitric oxide production and eNOS expression, supporting better blood vessel function.
- These findings suggest FCBP may help manage cardiovascular health by reducing inflammation and improving blood circulation. Further research is needed to confirm these effects in humans.

Globally, cardiovascular diseases related to blood circulation, such as hypertension, angina, myocardial infarction, and thrombosis, are increasingly prevalent, attributed to higher blood cholesterol levels due to rapid urbanization, economic growth, and shifts toward Western dietary and lifestyle patterns (1, 2). These trends are evident not only in specific regions like Korea but also across various countries, reflecting a widespread public health concern (3, 4). Hyperlipidemia, especially elevated low-density lipoprotein (LDL)-cholesterol levels, is a major risk factor for these conditions, significantly influencing their morbidity and mortality rates (5). This global perspective underscores the urgency in addressing cardiovascular health on a broader scale.

Under normal physiological conditions, the human body maintains a balance between blood coagulation and dissolution, preventing the formation of blood clots during circulation (6–8). However, this balance can be disrupted by excessive activation of blood coagulation factors or increased platelet aggregation, leading to thrombosis and embolism (9–11). These events can result in severe cardiovascular complications such as myocardial infarction and stroke (12, 13), with risk factors including poor diet, lifestyle habits, hyperlipidemia, hypertension, and diabetes (14, 15).

Current treatments for thrombotic diseases involve the use of anticoagulants (e.g. warfarin), antiplatelet agents (e.g. aspirin), and antithrombotic drugs (e.g. urokinase, t-PA) (16–18). Additionally, statin-type cholesterol-lowering drugs are recommended to reduce blood viscosity (19). However, these treatments are associated with adverse effects, such as bleeding, skin necrosis, and gastrointestinal complications. This highlights the need for alternative strategies, particularly functional foods that can prevent cardiovascular diseases at a fundamental level (20, 21).

Among potential candidates, hesperidin, a flavanone glycoside predominantly found in citrus peels, stands out despite its limited use due to low water solubility (22, 23). It is converted to hesperetin in the gastrointestinal tract and shows various biological activities, including vasodilation and antihypertensive, antithrombotic, anti-inflammatory, antilipidemic, and antioxidant properties in experimental models (24–30). However, the effects of hesperidin on endothelial function in humans have yielded mixed results, necessitating further research (31–33).

The fermented citrus bioconversion product (FCBP), the subject of this study, is a bioflavonoid formulation containing water-soluble hesperetin phosphate, which offers enhanced bioavailability and phytoactivity compared to non-phosphorylated counterparts (34–36). Previous studies have demonstrated its superior water solubility and physicochemical properties, resulting in increased plasma exposure and bioavailability in rat models (37, 38). This study aims to evaluate the effectiveness of non-toxic doses of FCBP in improving blood circulation-related functions using cell and animal test models, providing insights into its potential as a therapeutic agent for cardiovascular diseases.

## Materials and methods

### Preparation of FCBP

FCBP (RenoCidin™) was obtained from Vitech Co., Ltd. (Wanju, Korea) and was originally sourced from Hughes Biotech Co., Ltd. (Taipei City, Taiwan) for use in these experiments (34). Briefly, FCBP was prepared by cultivating *Bacillus subtilis* BCRC80517 with hesperetin in a nutrient broth medium for 48 h at 37°C, resulting in a 95% conversion of hesperetin to hesperetin phosphate derivates. The fermented broth was centrifuged to remove cells, and hesperetin phosphate derivates were extracted from the supernatant using ethyl acetate, followed by purification through a C-18 column. The fraction containing hesperetin 7-O-phosphate and hesperetin 3′-O-phosphate was collected, concentrated, and lyophilized to produce a powder with 95% purity, as confirmed by high-performance liquid chromatography (HPLC). The resulting lyophilized powder was used for further studies.

### Ethical statement and animal handling

The experimental protocol involving animals was approved by the Sahmyook University Animal Experiment Ethics Committee, adhering to the specified guidelines (WKU14-90). For the study, 60 male C57BL/6J mice, aged 8 weeks, were procured from Raon Bio (Yongin, Korea). The housing conditions for these animals were carefully regulated, with room temperature maintained at 23 ± 2°C and humidity levels kept between 50 and 60%. The light-dark cycle was set at 12-h intervals to simulate natural conditions. A preliminary acclimatization period of 1 week was provided to the animals before initiating experimental procedures, allowing them to adjust to the laboratory environment and minimize stress-related variables that could affect the study’s outcomes.

### Experimental design, treatment, and high-fat (HF) diet-induced hyperlipidemia induction

**Fig. 1:**
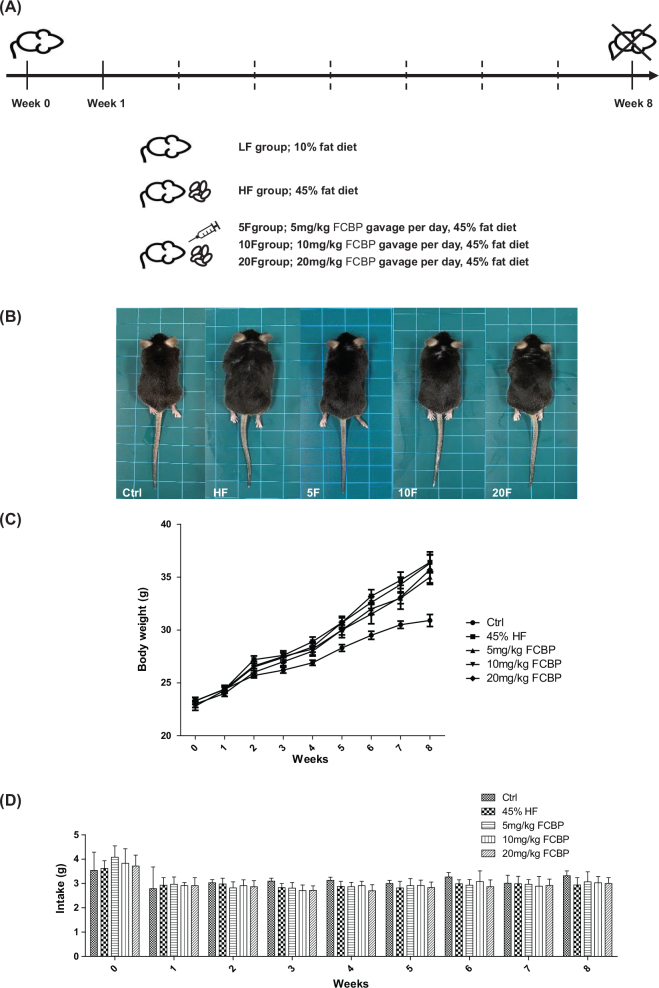
Experimental overview and impact of fermented citrus bioconversion product (FCBP) on body weight and diet intake in mice. (A) Schematic of experimental design. (B) Visual comparison of mice sizes. (C) Body weight progression over 8 weeks. (D) Food consumption tracked over the same period. C57BL/6 male mice were divided into 5 groups (*n* = 12 each) and treated with 0, 5, 10, or 20 mg/kg FCBP daily. Data expressed as mean ± SEM, analyzed by one-way analysis of variance (ANOVA) with Bonferroni’s multiple comparison test.

Following a 1-week acclimatization period, 60 mice were randomly allocated into five distinct experimental groups, each consisting of 12 animals (Fig. 1A). Over the course of an 8-week trial, the groups were subjected to different dietary and treatment regimens. The dietary groups included: (1) Low Fat (LF) diet group, receiving a diet comprising 10% kcal from fat; (2) High Fat (HF) diet group, receiving a diet with 45% kcal from fat; and three treatment groups wherein the HF diet was supplemented with FCBP at varying concentrations – (3) HF + 5 mg/kg FCBP (HF + 5F), (4) HF + 10 mg/kg FCBP (HF + 10F), and (5) HF + 20 mg/kg FCBP (HF + 20F). FCBP was administered once daily via oral gavage, dissolved in distilled water at the respective concentrations for each treatment group, ensuring consistent dosing. The mice were monitored weekly for body weight and food intake. Blood samples were collected at the end of the study (Week 8) for biochemical analysis, including serum nitric oxide (NO) levels and activated partial thromboplastin time (aPTT). Thoracic aorta tissues were harvested at the end of the study for histological and immunohistochemical analyses. Each experiment used 6–8 mice per group to ensure sufficient statistical power for detecting differences between groups.

### Blood and aorta collection methodology

Upon the completion of the experimental duration, the mice were anesthetized using isoflurane for humane handling. Blood samples were then collected directly from the heart using a sterile syringe. To prevent coagulation, the blood was immediately mixed with a 3.8% solution of sodium citrate as an anticoagulant, maintaining a ratio of 9 parts blood to 1 part sodium citrate. Following this, the blood samples were centrifuged at 3,000 × g, allowing for the separation of blood cells from the plasma. The isolated blood cells were utilized for the analysis of the interaction between oxidized red blood cells and annexin V. Meanwhile, the plasma was earmarked for the measurement of NO levels and for conducting aPTT analysis. Additionally, the thoracic aorta was carefully extracted, rinsed with phosphate-buffered saline (PBS), and preserved in 4% formalin for subsequent histological examinations.

### Measurement of NO in mouse plasma

The concentration of NO in the mouse plasma was quantified using the Griess Reagent System. This method involves the detection of nitrite (NO^2-^) as a stable end product of NO oxidation. The assay was conducted by mixing 100 µL of the collected mouse plasma with 100 µL of Griess reagent, composed of 1% sulfanilamide and 0.1% naphylethylenediamine dihydrochloride in 2.5% phosphoric acid. This mixture was then incubated for 5 min in a 96-well plate. The absorbance of the resultant solution was measured at 550 nm using a spectrophotometer. To accurately quantify the NO levels, a standard curve was established using varying concentrations of sodium nitrite.

### Measurement of aPTT

The aPTT, an important measure of the blood coagulation pathway, was assessed using a specific aPTT reagent (APTT-SP-0020006300, Instrumentation Laboratory). For this assay, the mouse plasma samples were mixed with the aPTT reagent according to the manufacturer’s instructions. This mixture was then analyzed using a blood coagulation analyzer, ACL9000 (Instrumentation Laboratory). The analyzer measured the time taken for the plasma to form a clot, which is the aPTT.

### Fluorescence-activated cell sorting (FACS) analysis

To assess the binding of oxidized red blood cells to annexin V, fluorescence flow cytometry was employed. This analysis was performed using the FITC Annexin V Apoptosis Detection Kit I (BD 556547, Pharming™, USA). The procedure began with washing the separated blood cells with PBS. The cell concentration was then adjusted to 1 × 10^5^ cells/mL using the kit’s binding buffer. Subsequently, the cells were stained by incubating them with a mixture of the binding buffer and FITC-labeled annexin V for 15 min at room temperature. Post-staining, the extent of annexin V binding to the cells was quantified using a flow cytometer (CytoFLEX, Beckman Coulter, USA). This method allowed for the precise detection and analysis of early apoptotic events in the blood cells, marked by the externalization of PS and its subsequent binding to annexin V.

### Histological examination of thoracic aorta

The thoracic aortas, previously fixed in 4% formalin, underwent a detailed histological examination. Initially, the aortas were rinsed with water to eliminate any residual formalin. They were then dehydrated through a graded ethanol series, starting from 60% and gradually increasing to 100% ethanol, followed by a xylene treatment for clearing. The tissues were then infiltrated with paraffin, and blocks were prepared by embedding the aortas in paraffin. Thin sections of 5 µm thickness were obtained using a rotary microtome (HistoCore AUTOCUT R, Leica, Germany). These sections were mounted on glass slides and air-dried at room temperature. For deparaffinization, the slides were treated with xylene, followed by rehydration through graded ethanol solutions (100, 80, and 70%). Subsequent to rehydration, the tissue sections underwent staining. Nuclear staining was carried out using Harris Hematoxylin solution (Abcam, USA), and cytoplasmic details were highlighted using 1% Eosin Y solution (Abcam, USA), culminating in a standard Hematoxylin and Eosin (H&E) staining process. Finally, the stained sections were observed under an optical microscope at a 400× magnification. Images were captured and documented using Axiovision 4 Imaging/Archiving software (Carl Zeiss, Germany).

### Immunohistochemistry (IHC) on thoracic aorta sections

Immunohistochemical analysis was performed on 5 μm thick sections obtained from the thoracic aorta paraffin blocks. Following deparaffinization and hydration, antigen retrieval was achieved by heating the tissue sections in 10 mM sodium citrate buffer (pH 6.0) at room temperature for 30 min. To block the activity of endogenous peroxidases, the sections were treated with a 3% hydrogen peroxide solution for 10 min. The sections were then washed with distilled water and incubated with 1% BSA (Bovine Serum Albumin, GenDEPOT, USA) in PBST for 10 min, followed by a 30-min incubation in 5% BSA in PBST. These steps were crucial to prevent non-specific antibody binding. Primary antibodies against ICAM-1 (SC-8439, Santa Cruz, USA) and VCAM-1 (SC-13160, Santa Cruz, USA) were applied at a dilution of 1:100 and incubated at room temperature for 2 h. Following primary antibody incubation, the sections were washed and then incubated with a secondary antibody, Anti-mouse IgG goat (F0257, Sigma, USA), diluted 1:200 for 30 min at room temperature. After a final washing step, the sections were mounted with a suitable medium for preservation. The immunostained sections were observed under a light microscope at a magnification of 400x. Images were captured and archived using Axiovision 4 Imaging/Archiving software (Carl Zeiss, Germany).

### Cell culture

EA.hy926 endothelial cells (ATCC CRL-2922, USA) were maintained in Dulbecco’s Modified Eagle Medium (DMEM; WELGENE Inc., Korea) supplemented with 10% heat-inactivated fetal bovine serum (FBS) and 1% penicillin/streptomycin. The cells were incubated at 37°C in a 5% CO_2_ environment (Thermo Scientific, USA).

### Cell viability assay

Cell viability was assessed using the 3-(4,5-dimethylthiazol-2-yl)-2,5-diphenyl-tetrazolium bromide (MTT; MT1036, Georgiachem, USA) assay. EA.hy926 cells were plated in 96-well plates at 2 × 10^4^ cells/well in 1% FBS DMEM and incubated for 24 h. Cells were then pretreated with 20 μg/mL FCBP for 1 h, followed by 10 ng/mL Tumor necrosis factor-α (TNF-α) (Peprotech, USA) treatment for 24 h. Post-incubation, cells were treated with 0.25 mg/mL MTT solution for 3 h. The formazan crystals formed were dissolved in 100 μL of dimethyl sulfoxide (DMSO; DUKSAN, Korea), and absorbance was measured at 570 nm using a microplate reader (Synergy H1, BioTek, USA).

### NO measurement

NO levels in cell culture media were quantified using the Griess Reagent System. Cells treated similarly to the cell viability assay were used. Post-incubation, 100 μL of supernatant was mixed with an equal volume of Griess reagent [1% sulfanilamide and 0.1% naphylethylenediamine dihydrochloride in 2.5% phosphoric acid] and incubated for 5 min. Absorbance was measured at 550 nm using a microplate reader (Synergy H1, BioTek, USA).

### Western blot analysis

For the analysis, EA.hy926 cells were cultured in 60 mm dishes at a density of 1 × 10^6^ cells/dish in DMEM containing 1% FBS. After 24 h of incubation, cells were pretreated with 20 μg/mL FCBP for 1 h, followed by stimulation with 10 ng/mL TNF-α (Peprotech, USA) for either 2 or 6 h. Post-treatment, cells were washed with PBS and lysed in 300 μL of lysis buffer. The lysates were centrifuged at 13,000 rpm for 10 min to remove cellular debris, and the supernatant containing proteins was collected. The protein concentrations were quantified using the BCA Protein Assay Kit (Thermo Scientific, USA), with BSA serving as the standard. Samples were prepared by mixing with Sodium Dodecyl Sulfate (SDS)-loading buffer in a 4:1 ratio and then denatured at 100°C for 5 min. Equal amounts of protein from each sample were loaded onto 6–10% SDS-polyacrylamide gels for electrophoresis. Following separation, proteins were transferred to Polyvinylidene fluoride (PVDF) membranes (Immobilon-P, Millipore). The membranes were blocked in 5% skim milk in Tris Buffered Saline with Tween 20 (TBST) (10 mM Tris, 150 mM NaCl, and 0.05% Tween 20) to prevent non-specific binding. Primary antibodies for ICAM-1 (SC-8439, Santa Cruz, USA), VCAM-1 (SC-13160, Santa Cruz, USA), endothelial nitric oxide synthase (eNOS) (#9572, Cell Signaling, USA), p-eNOS (#9571, Cell Signaling, USA), and β-actin (SC-47778, Santa Cruz, USA) were incubated with the membranes at a dilution of 1:1,000 overnight at 4°C. Subsequently, membranes were incubated with horseradish peroxidase (HRP)-conjugated secondary antibodies (Goat anti-Mouse IgG (H + L)-HRP, SA001-500, GenDEPOT, USA; Goat anti-Rabbit IgG(H + L)-HRP, SA002-500, GenDEPOT, USA) at a dilution of 1:10,000 at room temperature for 1 h. The protein bands were visualized using an enhanced chemiluminescence system (GE Healthcare, Menlo Park, CA, USA) and captured with a KwikQuant Imager (Kindle Biosciences LLC, CT, USA). The intensity of the bands was quantified using ImageJ software for relative protein expression analysis.

### Statistical analysis

All data collected from the experiments were statistically analyzed and are presented as the mean ± standard error of the mean (SEM), with error bars included in the figures to indicate variability and repeatability. To assess the significance of differences among experimental groups, a one-way Analysis of Variance (ANOVA) was performed using the Statistical Package for the Social Sciences (SPSS), version 23.0 (SPSS Inc., USA), followed by Bonferroni’s multiple comparison test. The analysis included multiple biological replicates, and the specific sample sizes (n) for each group were indicated in the figure legends. Data were considered statistically significant at *P < 0.05 compared to the control group.

## Results

### Assessment of body weight and food consumption

Figures 1B, 1C, and 1D illustrate the changes in body weight and food intake of the mice over the course of the experimental period. Mice fed a HF diet showed a significant increase in body weight compared to those on a normal diet, with the HF diet group displaying a marked weight gain starting from the second week of the experiment. In contrast, groups treated with the FCBP in addition to the HF diet demonstrated a dose-dependent reduction in weight gain: the 10 mg/kg FCBP group showed significant weight reduction from the 5th week, the 5 mg/kg group from the 6th week, and the 20 mg/kg group from the 8th week (*P* < 0.05). Despite these differences in weight gain, no significant differences in food intake were observed among the groups. The changes described in Fig. 1 highlight the potential effect of FCBP in mitigating weight gain associated with a HF diet.

### NO concentration in mouse serum

**Fig. 2:**
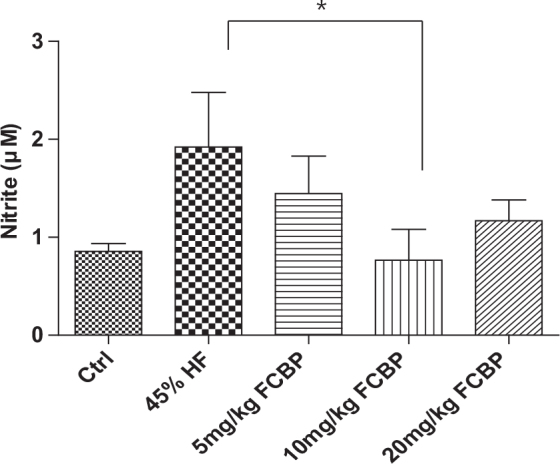
Serum nitrite levels as an indicator of nitric oxide (NO) production in mice. Following an 8-week diet regimen, serum nitrite concentrations were measured in mice administered with 0, 5, 10, or 20 mg/kg fermented citrus bioconversion product (FCBP) daily. Data expressed as mean ± SEM (standard error of the mean) (*n* = 6), analyzed by one-way analysis of variance (ANOVA) with Bonferroni’s multiple comparison test, *P < 0.05 compared to the high-fat diet group.

Figure 2 shows the serum NO levels measured in the experimental mice. The control group recorded an NO concentration of 0.86 ± 0.05 μM. In the HF diet group, NO levels increased to 1.92 ± 0.3 μM. For the groups receiving FCBP alongside the HF diet, the NO concentrations were as follows: 1.45 ± 0.22 μM for HF + 5F, 0.77 ± 0.18 μM for HF + 10F, and 1.17 ± 0.12 μM for HF + 20F. Notably, the HF diet group showed a significant elevation in serum NO levels compared to the control group. Among the FCBP treated groups, the HF + 10F group exhibited a significant reduction in serum NO levels compared to the HF group (*P* < 0.05).

### Analysis of aPTT in different dietary groups

**Fig. 3:**
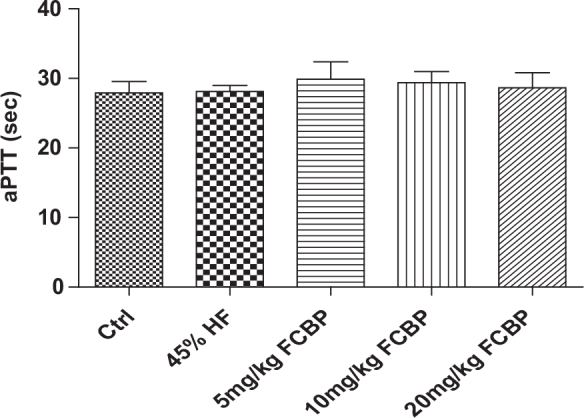
Evaluation of activated partial thromboplastin time (aPTT) in mice serum. After 8 weeks on respective diets, aPTT was measured in mice receiving 0, 5, 10, or 20 mg/kg fermented citrus bioconversion product (FCBP) daily. Data expressed as mean ± SEM (standard error of the mean) (*n* = 6), analyzed by one-way analysis of variance (ANOVA) with Bonferroni’s multiple comparison test.

Figure 3 details the aPTT for each experimental group. The average aPTT was 27.93 ± 0.93 seconds for the control group and 28.10 ± 0.50 s for the HF diet group. In the groups receiving FCBP along with the HF diet, the aPTT values were as follows: 29.87 ± 1.45 s for HF + 5F, 29.37 ± 0.93 s for HF + 10F, and 28.67 ± 1.24 s for HF + 20F. There was no statistically significant difference in aPTT between the groups. All groups showed aPTT levels within the normal range, with a slight tendency toward a reduction in a concentration-dependent manner in the groups supplemented with FCBP.

### PS exposure in mouse red blood cells

**Fig. 4:**
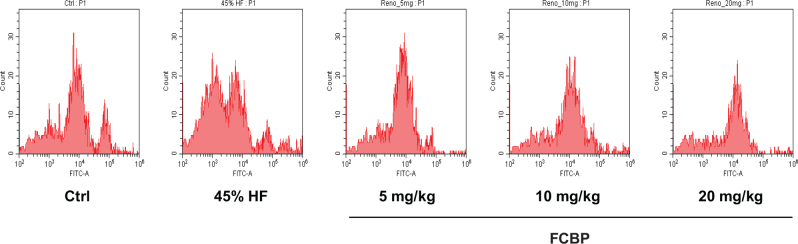
Annexin-V flow cytometry analysis for apoptosis in red blood cells of mice. Following an 8-week dietary regimen, apoptosis levels assessed in mice receiving varying doses of fermented citrus bioconversion product (FCBP). The plot shows Annexin V binding in non-oxidized and oxidized red blood cells (RBCs), highlighting apoptotic cells in the high-fat diet group compared to controls.

Figure 4 presents the results of the FACS analysis, focusing on PS exposure on mouse red blood cells. The data indicate an increased PS exposure in red blood cells from the HF diet group compared to the control group. Notably, in the groups treated with FCBP, a concentration-dependent decrease in PS exposure on red blood cells was observed compared to the HF diet group.

### CAM expression in thoracic aortic tissue

**Fig. 5:**
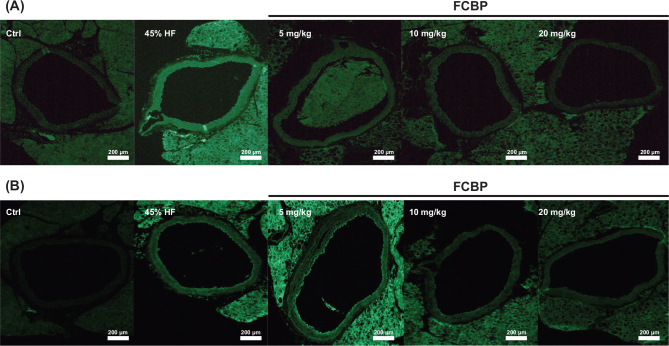
Immunohistochemical staining for VCAM-1 (A) and ICAM-1 (B) in mouse aorta tissue. Mice were fed different diets for 8 weeks and treated with 0, 5, 10, or 20 mg/kg fermented citrus bioconversion product (FCBP) daily. Staining intensity reflects changes in the expression of cell adhesion molecules in the aortic tissue.

Figure 5 illustrates the results of immunohistochemical staining for cell adhesion molecules (CAMs) such as ICAM-1 and VCAM-1 in thoracic aortic tissue sections from mice fed a HF diet. This analysis aimed to investigate the early stages of atherosclerosis. The results showed an increase in the expression of these CAMs in the thoracic aorta following stimulation with a HF diet. Moreover, a significant decrease in the expression of these molecules was observed in the thoracic aorta of mice treated with various concentrations of FCBP.

### Cell viability in EA.hy926 cells treated with TNF-α and FCBP

**Fig. 6:**
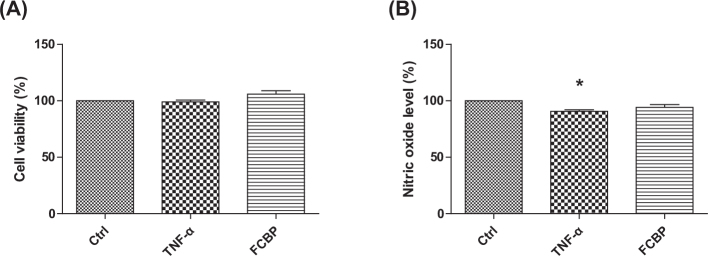
Cell viability and nitric oxide (NO) production in EA.hy926 cells treated with fermented citrus bioconversion product (FCBP). (A) Cell viability assessed by MTT assay in cells treated with 20 µg/mL FCBP and 10 ng/mL TNF-α for 24 h. (B) Nitrite concentration as an indicator of NO production under the same treatment conditions. Data expressed as mean ± SEM (standard error of the mean) (*n* = 3), analyzed by one-way analysis of variance (ANOVA) with Bonferroni’s multiple comparison test, *P < 0.05 compared to the control group.

Figure 6A illustrates the results of the cytotoxicity test for EA.hy926 cells treated with 10 ng/mL TNF-α and 20 μg/mL FCBP. The data show no significant change in cell survival rate compared to the control group at these concentrations, indicating the absence of cytotoxic effects under the tested conditions.

### NO levels under TNF-α and FCBP treatment

Figure 6B shows the measurement of NO secretion in EA.hy926 cells. The cells treated with TNF-α exhibited a reduction in NO levels to 90.75 ± 1.35% relative to the control. In contrast, cells treated with FCBP displayed NO levels at 94.19 ± 2.53%, aligning more closely with the control group’s levels, but without a statistically significant difference from the TNF-α treated group.

### CAM levels in response to TNF-α and FCBP

**Fig. 7:**
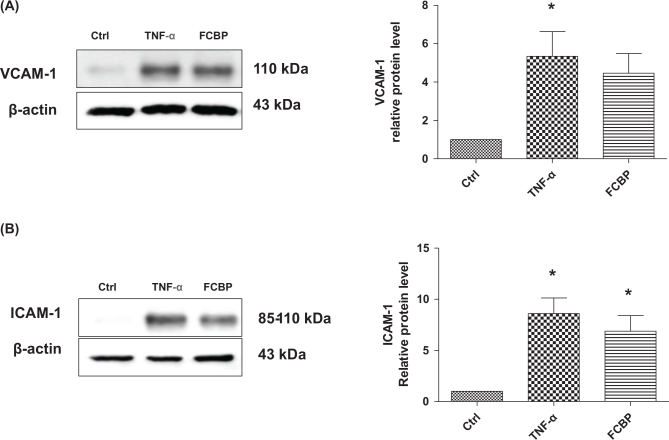
Influence of fermented citrus bioconversion product (FCBP) on TNF-α-induced VCAM-1 and ICAM-1 protein expression in EA.hy926 cells. Cells were pre-treated with 20 µg/mL FCBP for 1 h and then incubated with 10 ng/mL TNF-α for 6 h. Proteins were analyzed by SDS-PAGE and Western blotting for VCAM-1 (A) and ICAM-1 (B), with β-actin as a loading control. Densitometric ratios of proteins relative to β-actin are shown. Data expressed as mean ± SEM (standard error of the mean) (*n* = 3), analyzed by one-way analysis of variance (ANOVA) with Bonferroni’s multiple comparison test, *P < 0.05 compared to the control group.

Figures 7A and 7B delineate the expression levels of CAMs in EA.hy926 endothelial cells. VCAM-1 levels were observed to be the highest in the TNF-α treated group, measuring 8.21 ± 1.86. In the FCBP group, VCAM-1 levels showed a decrease to 7.77 ± 1.85, though this reduction was not statistically significant. Similarly, ICAM-1 levels were the highest in the TNF-α group, recorded at 8.60 ± 1.52, which was significantly higher than the control group (*P* < 0.05). The FCBP treated cells exhibited a lower level of ICAM-1 at 6.88 ± 1.52, compared to the TNF-α group, but this difference was not statistically significant.

### eNOS levels in EA.hy926 cells under TNF-α and FCBP treatment

**Fig. 8:**
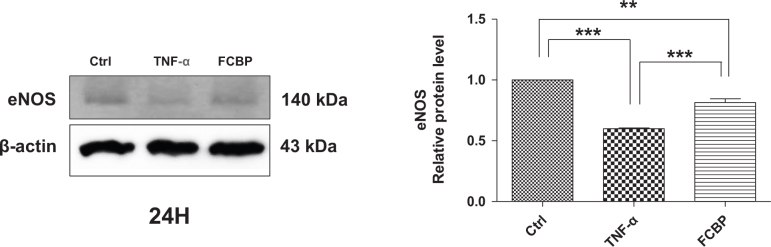
Effects of fermented citrus bioconversion product (FCBP) on TNF-α induced endothelial nitric oxide synthase (eNOS) protein expression in EA.hy926 cells. Pre-treatment with 20 µg/mL FCBP for 1 h followed by 10 ng/mL TNF-α for 24 h. Western blot analysis conducted for eNOS with β-actin as a loading control. Relative protein expression ratios over β-actin shown. Data expressed as mean ± SEM (standard error of the mean) (*n* = 3), analyzed by one-way analysis of variance (ANOVA) with Bonferroni’s multiple comparison test, **P < 0.01, ***P < 0.001 compared to the control group.

Figure 8 reports the expression levels of eNOS, a known anti-inflammatory marker, in EA.hy926 endothelial cells. In the TNF-α treated group, eNOS expression was found to be at its lowest, with a level of 0.38 ± 0.12. The FCBP treated group exhibited a higher eNOS level at 0.58 ± 0.17 compared to the TNF-α group, although this increase was not statistically significant.

## Discussion

In this study, key findings from the *in vivo* model demonstrated that mice on a HF diet showed significant weight gain, which was mitigated in a dose-dependent manner by FCBP supplementation. Serum NO levels increased in the HF diet group but significantly decreased with FCBP treatment at 10 mg/kg. PS exposure on red blood cells, elevated in the HF group, was reduced with FCBP treatment, suggesting decreased procoagulant activity. Additionally, immunohistochemical analysis revealed increased expression of inflammatory markers ICAM-1 and VCAM-1 in the thoracic aorta of HF diet mice, which was significantly reduced by FCBP. All groups maintained normal aPTTs, indicating no adverse effects on blood coagulation. The *in vitro* data supported these findings by demonstrating that FCBP had no cytotoxic effects on EA.hy926 endothelial cells in the presence of TNF-α, a trend of increased NO production and eNOS expression with FCBP treatment, and reduced expression of CAMs ICAM-1 and VCAM-1. These *in vitro* results provide insight into the mechanisms by which FCBP may alleviate HF diet-induced changes, including inflammation and early signs of atherosclerosis. Together, these findings suggest that FCBP holds promise in managing HF diet-induced cardiovascular changes, reducing inflammatory markers, and mitigating the progression of early atherosclerosis.

In the context of obesity-induced complications, FCBP showed a promising effect in reducing body weight gain in mice on a HF diet, a benefit that aligns with substances like green tea extracts and omega-3 fatty acids, which are known for their anti-obesity and lipid-lowering effects (39–42). Interestingly, the reduction in body weight with FCBP was dose-dependent, offering a potential advantage in controlling diet-induced obesity.

In our study’s FACS analysis, focusing on PS exposure in mouse red blood cells, a significant finding emerged from the FCBP treatment group. Notably, there was a concentration-dependent reduction in PS exposure on red blood cells in comparison to those fed a 45% HF diet. This observation is particularly important as it suggests that FCBP may play a role in modulating the procoagulant activity of red blood cells, a key factor in vascular health and disease prevention. The diminished PS exposure following FCBP treatment underscores its potential in countering the pro-thrombotic state commonly associated with HF diets, thus enhancing our understanding of its therapeutic capabilities in managing blood circulation and mitigating cardiovascular risk. However, despite this promising finding, the impact of FCBP on aPTT was not statistically significant. This highlights a potentially limited effect on the coagulation pathway, especially when compared to more potent anticoagulants such as omega-3 fatty acids or garlic extracts, as documented in various studies (43–45).

aPTT is an essential screening test for detecting abnormalities in both intrinsic and common coagulation pathways and serves as a crucial indicator of antithrombotic activity. In mice, the normal aPTT range, indicating the clotting time, is typically between 20 and 30 s. The structure of red blood cell membranes, with sphingomyelin (SM) and phosphatidylcholine (PC) on the outside and PS and phosphatidylinositol (PI) inside, maintains an asymmetric distribution. Stimulation leads to the disruption of this asymmetry and the exposure of PS, which potentiates the coagulation cascade. By treating red blood cells with annexin V, which specifically binds to PS, we could measure their procoagulant activity. Therefore, the primary function of FCBP, particularly in a HF diet-induced arteriosclerosis model, is believed to involve the improvement of blood circulation through the inhibition of coagulation factors in the blood vessel wall.

The observed decrease in the expression of CAMs (ICAM-1 and VCAM-1) in endothelial cells treated with FCBP reflects the anti-inflammatory effects similar to those of natural compounds like curcumin and omega-3 fatty acids, known for their ability to downregulate these markers, thereby mitigating vascular inflammation (46–49). This effect is significant in the context of atherosclerosis and cardiovascular diseases, where endothelial dysfunction is a major contributor. Oxidative damage to vascular endothelium triggers blood vessel inflammation and leads to the attachment of monocytes to the endothelial lining, initiating the development of arteriosclerosis. The vascular endothelium is thus a crucial site in the pathogenesis of arteriosclerosis, where the interaction between monocytes and endothelial cells is mediated by CAMs like VCAM-1 and ICAM-1. During inflammation, the expression of these adhesion factors is upregulated, facilitating monocyte attachment to the endothelium and contributing to the progression of arteriosclerosis. Numerous domestic and international studies have demonstrated that in arteriosclerosis, increased expression of VCAM-1 and ICAM-1 leads to intercellular adhesion, exacerbating vascular stenosis and thrombus formation (50–53). The findings of this study, indicating that FCBP inhibits the expression of these CAMs, suggest its potential in effectively hindering the early development of arteriosclerosis.

The study’s observation of a trend toward increased NO production and eNOS expression with FCBP treatment, though not statistically significant, aligns with the effects of bioactive substances like resveratrol and quercetin. These compounds are documented to enhance NO production and eNOS activity, thereby improving vascular function (54–56). However, the impact of FCBP on these parameters seems modest compared to these substances, hinting at a potentially different action mechanism or pathway. Endothelial cells secrete NO to dilate blood vessels, and inhibition of eNOS can lead to endothelial dysfunction, vasoconstriction, inflammation, and thrombosis, critical in early arteriosclerosis stages. FCBP’s ability to increase eNOS-related protein expression suggests it may effectively inhibit early arteriosclerosis development by exerting an antithrombotic effect. NO plays a vital role in maintaining endothelial cell homeostasis, synthesized from L-arginine in endothelial cells. It diffuses into vascular smooth muscle cells, promoting relaxation through soluble guanylate cyclase activation. However, dietary fat significantly influences NO levels. Studies, including one on female rats fed a HF diet, indicate that such diets increase reactive oxygen species production, leading to reactive oxygen radicals. This results in reduced NO synthase activity and NO oxidation, inducing hypertension (57–60). While NO is essential for physiological functions like nerve transmission and immune response, excessive production can cause adverse effects like vasodilation, inflammation, and tissue damage. Hence, the role of FCBP in modulating NO production and eNOS activity, especially under HF dietary conditions, is of significant interest for its potential therapeutic benefits in cardiovascular health and disease prevention. While our study primarily focused on the effects of FCBP on NO production and eNOS expression, it is important to consider its potential free radical scavenging activities. Although specific assays for superoxide anion and hydroxyl radical scavenging were not conducted, the observed increase in NO production suggests an indirect antioxidant effect, as NO can neutralize ROS such as superoxide anions, thereby reducing oxidative stress. Previous studies have established that hesperetin and its derivatives possess significant free radical scavenging abilities (28), which implies that FCBP, with its enhanced bioavailability and interaction with biological systems, may also exhibit similar activities. Future research should include direct assessments of FCBP’s scavenging effects on superoxide anions and hydroxyl radicals to further elucidate its antioxidative potential and its role in protecting against oxidative stress in cardiovascular health.

The research findings on FCBP present intriguing insights into its potential role in improving blood circulation and mitigating the effects of a HF diet, drawing comparisons with existing bioactive substances known for similar benefits (61–65). One of the key observations was the absence of cytotoxic effects in EA.hy926 endothelial cells treated with FCBP. This aligns with studies on other flavonoids and citrus-based supplements, which also demonstrate a high safety profile and lack of toxicity at similar concentrations (66–68).

FCBP, a fermented citrus bioflavonoid, is derived through the microbial phosphorylation of hesperetin, primarily by *Bacillus subtilis* BCRC 80517 (34). This process significantly enhances the physico-chemical properties of hesperetin, particularly its water solubility and bioavailability, which are crucial for its biological efficacy in cardiovascular health management. The phosphorylation of hesperetin, leading to the formation of active constituents such as hesperetin 7-O-phosphate, not only improves solubility but also enhances bioactivity by facilitating greater interaction with biological membranes and enzymes. This enhancement is likely responsible for the observed increases in NO production and eNOS expression in this study, suggesting that FCBP may more effectively promote endothelial function compared to non-phosphorylated hesperetin. These findings underscore FCBP’s potential as a therapeutic agent in improving vascular health, with further research needed to fully elucidate its active constituents and broader therapeutic implications.

This study, while providing valuable insights, is not without its limitations. The modest changes in NO production and eNOS expression and the lack of significant impact on PS exposure and aPTT suggest that the effects of FCBP, although promising, might not be as pronounced as some established bioactive substances. While this study focused on evaluating key markers of endothelial inflammation, such as ICAM-1 and VCAM-1, as well as the effects on NO production and eNOS expression, it is important to acknowledge the role of C-reactive protein (CRP) as a critical marker of systemic inflammation. Unfortunately, due to the limitations of our study design and the exhaustion of blood samples, we were unable to measure CRP levels in this investigation. However, the observed reductions in ICAM-1 and VCAM-1 expression, along with the trends in increased NO production and eNOS expression, suggest that FCBP may have significant anti-inflammatory effects, which could be correlated with lower CRP levels. Future studies will aim to include CRP measurements to provide a more comprehensive assessment of FCBP’s anti-inflammatory potential and its broader implications for cardiovascular health. Additionally, the reliance on a single cell line and a specific animal model may limit the generalizability of the findings. Future research should focus on broader cell and animal models, including human clinical trials, to validate these results. Also, a deeper investigation into the molecular mechanisms underlying FCBP’s action would be beneficial. Furthermore, comparing its efficacy directly with other well-known bioactive compounds could provide a clearer understanding of its relative potency and potential applications in cardiovascular health management. This comprehensive approach would offer a more conclusive assessment of FCBP’s therapeutic value and its place in the broader context of cardiovascular disease prevention and treatment.

In summary, FCBP exhibits a profile that suggests its potential utility in improving blood circulation and mitigating diet-induced changes, akin to other known bioactive substances. Its effects on NO production, weight management, and inflammatory markers position it as a promising candidate for further exploration in the context of cardiovascular health. However, its modest effects on certain parameters, compared to more established bioactives, indicate the need for further studies to fully understand its mechanism and efficacy in blood circulation improvement.

## Conclusions

This study highlights the potential of FCBP in managing cardiovascular health, particularly under HF diet conditions. Our *in vivo* findings demonstrated that FCBP effectively mitigates weight gain in a dose-dependent manner and reduces inflammatory markers (ICAM-1 and VCAM-1) in endothelial cells, suggesting its role in managing diet-induced obesity and preventing early atherosclerosis. Although the effects on NO production, eNOS expression, PS exposure, and coagulation time (aPTT) were modest, these results suggest FCBP has a multifaceted influence on cardiovascular markers, with a notable anti-inflammatory effect.

Furthermore, the absence of cytotoxic effects on endothelial cells supports its safety profile, making it a potentially valuable bioactive compound for cardiovascular health management. While these findings are promising, further studies are necessary to clarify the mechanisms underlying FCBP’s effects, assess its long-term safety, and directly compare its efficacy with other well-known bioactive compounds. Continued research will provide deeper insights into FCBP’s potential therapeutic applications in blood circulation improvement and cardiovascular disease prevention.

## Data Availability

The data used to support of this study are included within the article.
